# Left ventricular stroke volume index predicts outcomes in asymptomatic chronic severe primary mitral regurgitation

**DOI:** 10.1186/s44348-026-00088-3

**Published:** 2026-08-24

**Authors:** Porntera Sethalao, Nithima Ratanasit, Decho Jakrapanichakul, Sasima Tongsai, Sirichai Jamnongprasatporn

**Affiliations:** 1 Division of Cardiology, Department of Medicine, Faculty of Medicine Siriraj Hospital, Mahidol University, Bangkok, Thailand; 2 Research Department, Faculty of Medicine Siriraj Hospital, Mahidol University, Bangkok, Thailand

**Keywords:** Mitral regurgitation, Left ventricular stroke volume index, Mitral valve surgery, Mitral valve intervention

## Abstract

**Background:**

Severe primary mitral regurgitation (MR) linked to substantial morbidity and mortality. However, racial differences impact the left ventricular size, which subsequently influence specific echocardiographic parameters. In this context, the left ventricular stroke volume index (LVSVi) may serve as a valuable prognostic indicator. This study aims to examine the association between LVSVi and a composite endpoint of all-cause mortality or mitral valve (MV) surgery.

**Methods:**

We conducted a retrospective study on asymptomatic chronic severe primary MR patients over a 5-year follow-up period.

**Results:**

Among 77 patients with asymptomatic severe primary MR (mean age, 56.9 years; 55 male patients, 71.4%), an increase of 1 mL/m^2^ in LVSVi, calculated from the left ventricular outflow tract area, was independently associated with an 8.2% reduction in the odds of reaching the composite endpoint of all-cause mortality or MV surgery (adjusted odds ratio, 0.918; 95% confidence interval, 0.856–0.986; *P*** = **0.018). The receiver operating characteristic curve for LVSVi demonstrated an area under the curve of 0.726 (*P*** = **0.002), with a threshold of ≤ 35 mL/m^2^ yielding a sensitivity of 81% and specificity of 50% for predicting the composite outcome. The 5-year event-free survival was significantly lower in patients with LVSVi ≤ 35 mL/m^2^ compared to those with LVSVi > 35 mL/m^2^ (22.9% vs. 67.0%; Breslow test, *P* < 0.001).

**Conclusions:**

In patients with asymptomatic chronic severe primary MR, LVSVi was the significant echocardiographic parameter influencing the outcomes of MV surgery and mortality. This study supported the hypothesis generation that LVSVi may serve as a prognostic marker in this specific population.

## Background

Severe primary mitral regurgitation (MR) is a progressive disease associated with significant morbidity and mortality [1–4]. Since the pathological changes in primary MR predominantly impact the mitral valve (MV) leaflets or apparatus, surgical or interventional procedures on the MV are essential for managing severe MR [5]. According to American and European guidelines for managing patients with severe primary MR, MV surgery is recommend in symptomatic individuals and asymptomatic patients with left ventricular (LV) systolic dysfunction, evidenced by LV ejection fraction (LVEF) of ≤ 60% and/or LV end-systolic diameter (LVESD) of ≥ 40 mm [6–9]. Moreover, MV surgery should be considered for asymptomatic patients with preserved LV function who exhibit atrial fibrillation (AF) due to MR, pulmonary hypertension, or significant left atrial (LA) dilation [7].

Racial differences impact the body and LV size, which subsequently influence specific echocardiographic parameters. Recent echocardiographic studies reveal that the average LV end-systolic volume in white individuals is 25.5% larger than in Asian individuals. Conversely, LVEF in white individuals is 2.2% lower compared to that of Asian individuals [10]. This emphasizes that standard guidelines may not apply to certain patients, particularly those with smaller LV sizes. As MR advances, LV outflow tract (LVOT) stroke volume decreases. Previous studies have shown that several LV stroke volume (LVSV) indices are linked to the need for MV surgery and mortality [11–13]. We hypothesized that a simple echocardiographic parameter, the LVSV index (LVSVi; defined as LVSV divided by body surface area), may provide prognostic value in asymptomatic patients with chronic severe primary MR.

This study aimed to investigate the association between the LVSVi and the composite endpoint of all-cause mortality and MV surgery in patients with asymptomatic chronic severe primary MR over a 5-year follow-up period, focusing on the population with LV sizes that deviate from standard guideline criteria.

## Methods

### Ethics statement

This study was approved by the Ethics Committee of Siriraj Hospital, Mahidol University (No. Si 286/2024). The requirement for informed consent was waived due to the use of deidentified data and the retrospective nature of the study.

### Study design and population

We retrospectively identified patients with asymptomatic chronic severe primary MR who underwent echocardiographic evaluation at the Echocardiography and Non-Invasive Testing Laboratory, Siriraj Hospital, between January 1, 2018, and December 31, 2019. Patients with acute primary MR, a history of MV replacement or repair, concomitant congenital heart disease, moderate to severe aortic valve disease, or pregnancy were excluded. Clinical history, baseline characteristics, echocardiographic findings, mortality data, and records of subsequent MV surgery were retrieved from electronic medical records. The primary endpoint was a composite of all-cause mortality and MV surgery occurring within 5 years after the index echocardiographic evaluation. Follow-up duration was defined as the interval from the date of the index echocardiography to the occurrence of the composite outcome or the last documented clinical follow-up.

### Echocardiographic data

Transthoracic echocardiography was performed using a Philips CVX system (Philips). The severity of MR was graded according to the American Society of Echocardiography guidelines for the assessment of valvular regurgitation [5]. The effective regurgitant orifice (ERO) and regurgitant volume (RVol) were calculated using the proximal isovelocity surface area (PISA) method and served as the primary parameters for defining the severity of MR. LV size, LVEF by the biplane Simpson method, LVOT diameter, and LVOT velocity–time integral (VTI) were measured in accordance with established echocardiographic guidelines [14]. LVSV was calculated by multiplying the LVOT cross-sectional area by the LVOT VTI. LVSV was then divided by body surface area to derive the LVSVi.

### Statistical analysis

Continuous variables were assessed for normality and are presented as mean** ± **standard deviation for normally distributed data, or as median with interquartile range (IQR) for non-normally distributed data. Categorical variables are expressed as counts and percentages. Comparisons between two groups were performed using the independent t-test for normally distributed continuous variables and the Mann–Whitney U-test for non-normally distributed variables. Categorical variables were compared using the chi-square test with Yates continuity correction or Fisher exact test, as appropriate.

Univariable and multivariable binary logistic regression analyses were performed to identify factors associated with the composite outcome. Variables with a P-value of < 0.10 in the univariable analysis were prespecified for inclusion in the multivariable model. Given the relatively limited sample size and number of outcome events, the number of covariates included in the final model was intentionally restricted to minimize the risk of model overfitting. Results are reported as odds ratios (ORs) with corresponding 95% confidence intervals (CIs).

Receiver operating characteristic (ROC) curve analysis was performed to evaluate the discriminative performance of LVSVi, and the area under the curve (AUC) was calculated. The optimal cutoff value of LVSVi for predicting the composite outcome was determined based on the ROC analysis.

Time-to-event analyses were performed using the Kaplan–Meier method and Cox proportional hazards regression analysis. Differences in event-free survival between groups were compared using the Breslow (generalized Wilcoxon) test. Hazard ratios (HRs) with corresponding 95% CIs were reported.

All statistical analyses were two-tailed, and a *P*-value of < 0.05 was considered statistically significant. Analyses were performed using IBM SPSS ver. 27.0 (IBM Corp).

## Results

A total of 77 patients with asymptomatic severe primary MR were identified. Baseline characteristics stratified by LVSVi are presented in Table 1. The mean age was 56.9 years, and 55 patients (71.4%) were male. AF was observed in only six patients (7.9%), as only clinically documented AF confirmed by 12-lead electrocardiography was included. The median follow-up duration was 48 months (IQR, 20–66 months). Transesophageal echocardiography was performed in 23 patients (29.9%). The composite outcome occurred in 24 patients (31.2% including 3 deaths (12.5%) and 21 MV surgeries (87.5%). The median time from asymptomatic status to the composite outcome was 30.5 months. All the baseline clinical characteristics and echocardiographic parameters related to LVSVi are presented in Tables 1 and 2, respectively.

**Table 1:** Baseline clinical characteristics according to LVSVi

Characteristic	Overall (n = 77)	LVSVi		*P*-value
		≤ 35 mL/m^2^ (n = 17)	> 35 mL/m^2^ (n = 60)	
Age (yr)	56.9 ± 14.1	59.4 ± 14.9	56.2 ± 14.5	0.430
Sex				0.229
Male	55 (71.4)	10 (58.8)	45 (75.0)	
Female	22 (28.6)	7 (41.2)	15 (25.0)	
Body surface area (m^2^)	1.68 ± 0.18	1.66 ± 0.16	1.68 ± 0.19	0.682
Comorbidity				
Hypertension	42 (54.5)	10 (58.8)	32 (53.3)	0.900
Diabetes mellitus	8 (10.4)	2 (11.8)	6 (10.0)	> 0.999
Dyslipidemia	27 (35.1)	5 (29.4)	22 (36.7)	0.791
Coronary artery disease	1 (1.3)	1 (5.9)	0 (0)	0.221
Atrial fibrillation	6 (7.9)	2 (11.8)	4 (6.8)	0.611
Chronic kidney disease	8 (10.4)	2 (11.8)	6 (10.0)	> 0.999
Rheumatic heart disease	1 (1.3)	1 (5.9)	0 (0)	0.221
Medication				
Loop diuretic	5 (6.6)	2 (12.5)	3 (5.0)	0.282
ACE-I/ARB	34 (44.7)	6 (37.5)	28 (46.7)	0.710
β-Blocker	17 (22.4)	5 (31.3)	12 (20.0)	0.333

Values are presented as mean ± standard deviation or number (%)

ACE-I, angiotensin-converting enzyme inhibitor; ARB, angiotensin receptor blocker; LVSVi, left ventricular stroke volume index

**Table 2:** Baseline echocardiographic parameters according to LVSVi

Parameter	Overall **(**n = 77**)**	LVSVi		P-value
		≤ 35 mL/m^2^ (n = 17)	> 35 mL/m^2^ (n = 60)	
LVEF (%)	69.5 ± 5.4	70.5 ± 4.8	69.2 ± 5.6	0.381
LVESD (mm)	31.5 ± 4.4	30.5 ± 4.8	31.9 ± 4.3	0.251
LVESDi (mm/m^2^)	19.0 ± 3.0	18.6 ± 3.8	19.1 ± 2.8	0.631
LVEDV (mL)	139.2 ± 33.2	133.4 ± 32.2	141.1 ± 33.7	0.405
LVESV (mL)	40.8 ± 13.6	37.6 ± 14.0	41.8 ± 13.5	0.265
LAVi (mL/m^2^)	60.6 ± 21.6	69.4 ± 24.4	63.6 ± 49.2	0.054
ERO (mm^2^)	67.4 ± 24.8	81.2 ± 35.9	41.8 ± 13.5	0.010
RVol (mL)	110.4 ± 35.6	127.2 ± 42.2	105.7 ± 32.5	0.028
MV peak E velocity (cm/sec)	102.1 ± 27.7	100.9 ± 28.0	103.2 ± 27.7	0.800
E/e’ (medial)	14.6 ± 5.4	14.4 ± 5.9	14.7 ± 5.3	0.890
TAPSE (mm)	24.6 ± 4.2	24.6 ± 4.4	24.6 ± 4.3	0.968
RVSP (mmHg)	38.8 ± 13.0	41.7 ± 17.5	37.5 ± 11.2	0.286

Values are presented as mean ± standard deviation

ERO, effective regurgitant orifice; LAVi, left atrial volume index; LVEF, left ventricular ejection fraction; LVESD, left ventricular end-systolic dimension; LVEDV, left ventricular diastolic volume; LVESDi, left ventricular end-systolic dimension index; LVESV, left ventricular systolic volume; LVSVi, left ventricular stroke volume index; MV, mitral valve; RVol, regurgitant volume; RVSP, right ventricular systolic pressure; TAPSE; tricuspid annular plane systolic excursion

Univariable logistic regression analysis was conducted to identify factors associated with the composite outcome. The variables evaluated included age, sex, AF, LVSVi, LVEF, LVESD, LVESD index (LVESDi), LA volume index (LAVi), right ventricular systolic pressure, ERO, and RVol. Among these, LVSVi (*P*** = **0.004), LVESD (*P*** = **0.051), and LAVi (*P*** = **0.015) demonstrated potential association with the composite outcome at the prespecified threshold of P < 0.10. Variables that met the prespecified screening criterion were subsequently included in the multivariable logistic regression model. In the multivariable analysis, LVSVi emerged as the only independent predictor of the composite outcome, with an adjusted OR of 0.918 (95% CI, 0.856–0.986; *P*** = **0.018), as presented in Table 3. This indicates that for each 1 mL/m^2^ increase in LVSVi was associated with an 8.2% reduction in the odds of the composite outcome of all-cause mortality or MV surgery.

**Table 3:** Univariable and multivariable predictors of the composite outcome of all-cause mortality and mitral valve surgery

Factor	Univariable analysis		Multivariable analysis	
	Crude OR (95% CI)	*P*-value	Adjusted OR (95% CI)	*P*-value
LVSVi (mL/m^2^)	0.916 (0.863–0.972)	0.004	0.918 (0.856–0.986)	0.018
LVESD (mm)	1.097 (1.000–1.203)	0.051	1.068 (0.968–1.179)	0.189
LAVi (mL/m^2^)	1.018 (1.003–1.032)	0.015	1.007 (0.991–1.022)	0.394
Age (yr)	0.989 (0.962–1.017)	0.433		
Sex	1.268 (0.756–2.125)	0.368		
Atrial fibrillation	2.487 (0.734–8.426)	0.143		
LVEF (%)	0.952 (0.872–1.038)	0.264		
LVESDi (mm/m^2^)	1.077 (0.939–1.235)	0.287		
RVSP (mmHg)	1.020 (0.988–1.053)	0.227		
ERO (cm^2^)	1.008 (0.990–1.025)	0.388		
RVol (mL)	0.999 (0.987–1.012)	0.923		

Variables with *P* < 0.10 in univariable analysis (LVSVi, LVESD, and LAVi) were entered into the multivariable model. Due to the limited sample size and number of outcome events, additional clinically relevant variables that did not meet the prespecified screening criterion were not included in the final multivariable model to minimize overfitting

CI, confidence interval; ERO, effective regurgitant orifice; LAVi, left atrial volume index; LVEF, left ventricular ejection fraction; LVESD, left ventricular end-systolic dimension; LVESDi, left ventricular end-systolic dimension index; LVSVi, left ventricular stroke volume index; OR, odds ratio; RVol, regurgitant volume; RVSP, right ventricular systolic pressure

**Fig. 1:**
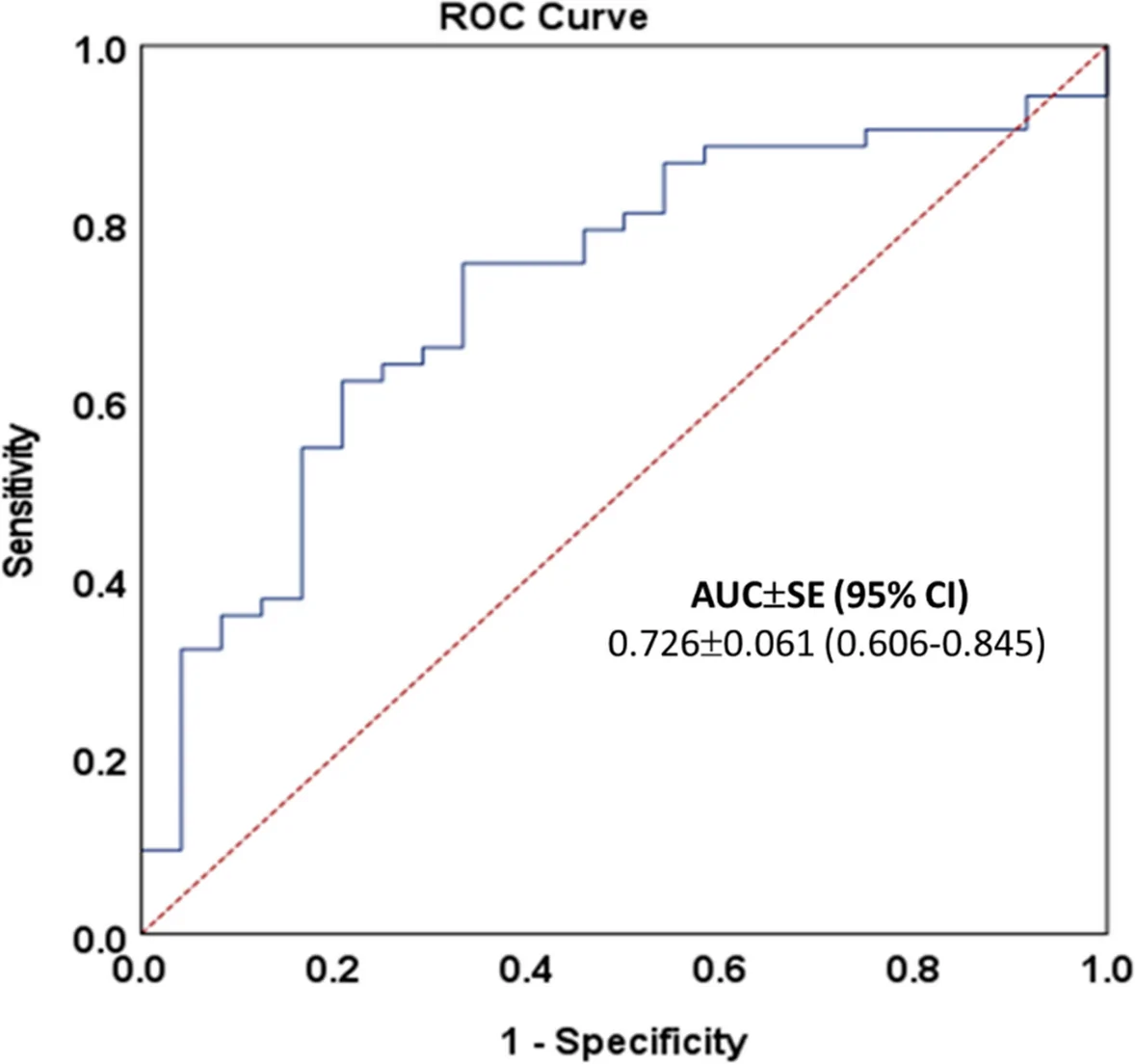
Receiver operating characteristic (ROC) curve for left ventricular stroke volume index (LVSVi) in predicting the composite outcome of all-cause mortality and mitral valve surgery. The area under the curve (AUC) was 0.726 (95% confidence interval [CI], 0.606–0.845; *p***= **0.002), indicating acceptable discriminative ability. SE, standard error

The discriminative performance of LVSVi in predicting the composite outcome was further evaluated using ROC curve analysis (Fig. 1). The AUC was 0.726 (95% CI, 0.606–0.845; *P*** = **0.002), indicating acceptable predictive accuracy. A cutoff value of 35 mL/m^2^ provided the optimal balance between sensitivity and specificity, yielding a sensitivity of 81% and a specificity of 50% for predicting the composite outcome. Thus, the final ROC analysis identified 35 mL/m^2^ as the best cutoff value for LVSVi.

**Fig. 2:**
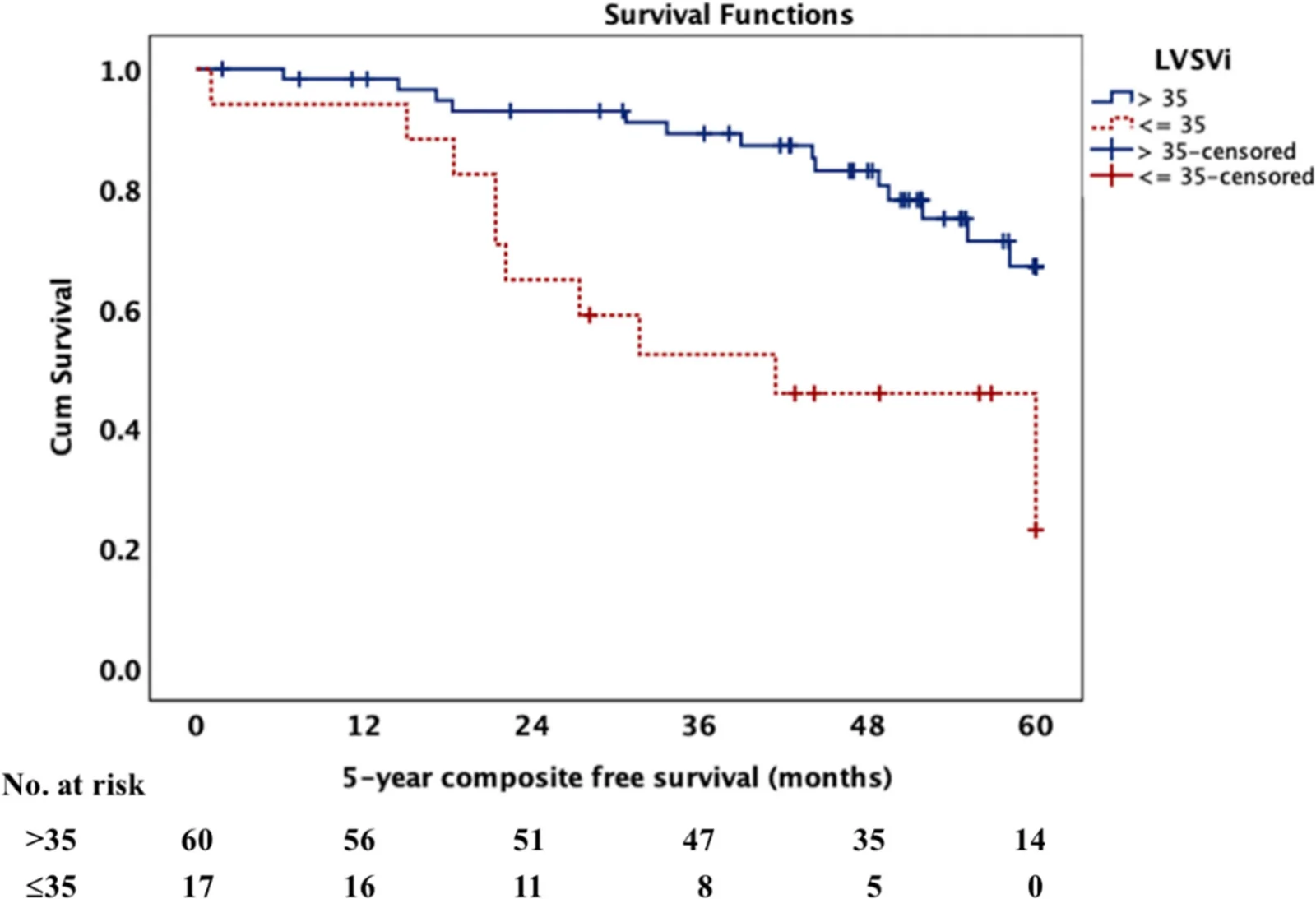
Kaplan–Meier curves for the 5-year event-free survival according to left ventricular stroke volume index (LVSVi). Patients were stratified into low (≤ 35 mL/m^2^) and normal (> 35 mL/m^2^) LVSVi groups. Event-free survival reflects freedom from the composite outcome of all-cause mortality and mitral valve surgery

Figure 2 illustrates the Kaplan–Meier curves for the 5-year composite event-free survival stratified by LVSVi. Patients with a low LVSVi (≤ 35 mL/m^2^) demonstrated significantly reduced event-free survival compared to those with a higher LVSVi (> 35 mL/m^2^). The difference in survival distributions between the two groups was statistically significant, as assessed by the Breslow test (*P* < 0.001). In unadjusted Cox proportional hazards analysis, patients with an LVSVi ≤ 35 mL/m^2^ had a 3.18-fold increased risk of experiencing the composite outcome of all-cause mortality or MV surgery (HR, 3.178; 95% CI, 1.332–7.582; *P*** = **0.009), compared with those with an LVSVi > 35 mL/m^2^.

Based on the baseline clinical characteristics and echocardiographic parameters, increased ERO and RVol were significantly associated with an LVSVi ≤ 35 ml/m^2^ (*P*** = **0.010 and *P*** = **0.028, respectively), which was in turn associated with a poorer composite outcome.

## Discussion

Our study identified LVSVi as the sole independent predictor of the composite endpoint of all-cause death and MV surgery in patients with asymptomatic chronic severe MR. Current guideline recommendations for MV intervention in this population are based on limited studies that primarily included white patients [4, 6–8]. Tribouilloy et al. [8] reported that a LVESD greater than 40 mm was associated with an increased risk of mortality both under medical management and following MV surgery. The average LVESD in their study was 13.6% larger than that observed in our cohort. Furthermore, the LVEF was 6% lower compared to our findings. If current guideline criteria were applied to our cohort, which is characterized by smaller LV dimensions, MV intervention would likely be delayed, potentially resulting in increased morbidity and mortality. This is particularly concerning given that the Asian population accounts for approximately 60% of the global population [15]. Our study highlights a previously overlooked yet sizable population. Notably, we found that other guideline-recommended parameters for MV surgery (such as pulmonary hypertension and AF) were not predictive of composite outcomes in our cohort. We hypothesize that this discrepancy may be attributed to differences in study populations, as the referenced study had an average LVESD that was 15.9% larger than ours and included a symptomatic population accounting for 35% of its subjects [16].

Several studies have demonstrated the utility of various LVSVi [11–13]. Dupuis et al. [11] reported that forward LVEF, calculated as 100 × (forward stroke volume/LV end-diastolic volume), was associated with the composite endpoint of MV surgery and death. Notably, their results also demonstrated that an LVSVi less than 35 mL/m^2^ was significantly associated with adverse outcomes. However, their study initially included patients with all degrees of MR severity, with 80% having mild to moderate MR whereas our study exclusively included patients with severe MR. Furthermore, nearly all cases in their study (99%) were due to MV prolapse, which poses challenges in accurately grading severity, particularly in cases of nonholosystolic MR. In contrast, our cohort included flail or ruptured chordae in 59% of patients, while only 20.5% had prolapse, providing a broader representation of severe MR etiology. Another study by Petolat et al. [13] included 198 patients with MV prolapse who underwent MV repair and found that LVOT VTI and LVSVi were associated with postoperative LV dysfunction. However, this study was limited to patients with MR due to MV prolapse. In addition, their primary outcome was postoperative LVEF, a surrogate endpoint rather than a clinical outcome. Notably, 51.5% of their patients had a class I indication for surgical intervention, either due to symptoms or echocardiographic parameters. In contrast, our study only included asymptomatic patients, with only 2 out of 78 patients (2.5%) meeting class I criteria for intervention based on an LVESD > 40 mm.

In the context of chronic aortic regurgitation, previous studies have suggested that women experience higher mortality rates than men, likely due to their smaller LV size, which contributes to delayed surgical intervention [17]. Therefore, LVESDi has been used to guide the need for aortic valve intervention, particularly in patients with smaller body size. A similar approach may be applicable in cases of MR among individuals with small body size. Unfortunately, our study found no significant association between LVESDi and clinical outcomes. This aligns with the findings of Petolat et al. [13], where LVESDi was associated with postoperative LVEF in univariate analysis but lost significance in multivariate analysis.

Taken together, our study included a pure cohort of asymptomatic patients with chronic severe primary MR, none of whom met class I indications for MV surgery. We found that LVSVi was significantly associated with the composite outcome of death and MV surgery, particularly in patients with LV sizes that differed from current guideline thresholds. This important finding is hypothesis-generating and warrants further investigation in diverse populations and should, at a minimum, prompt primary care physicians to actively monitor patients.

### Limitations

This study has several limitations. First, its retrospective, single-center cohort design makes it susceptible to unmeasured confounding factors that could not be fully adjusted for and may have influenced the observed outcomes. Second, although MR was graded using a multiparametric approach, the PISA method was the primary technique used to determine ERO and RVol. Given the predominance of eccentric jets in our cohort, nearly 80% of cases involved ruptured chordae or valve prolapse, there is a potential for PISA overestimation due to jet impingement on the LV wall, which could lead to overestimation of both ERO and RVol. Third, LV volumes were measured using 2D echocardiography, which may significantly underestimate volumes compared to three-dimensional methods [14]. Fourth, due to the relatively small sample size and limited number of clinical events, the statistical power was limited and only a restricted number of covariates could be included in the multivariable model, thereby increasing the possibility of residual confounding. Fifth, according to the low mortality rate, the primary outcome was predominantly driven by MV surgery rather than mortality, which may represent a less robust clinical endpoint. Sixth, an unexpectedly low rate of AF in the present study of chronic severe MR patients raised the concern that AF may be underdetected and may lead to a lack of prognostic significance of AF in this study. According to several limitations as mentioned above, our data supported the hypothesis generation that LVSVi may be utilized as a prognostic marker in this specific subgroup of patients. However, this parameter cannot replace or demonstrate superiority over the existing guideline-recommended parameters.

## Conclusions

We demonstrated that in patients with asymptomatic chronic severe primary MR who had different body and LV size from the previous guideline, LVSVi was the significant echocardiographic parameter associated with MV surgery outcomes and mortality. We proposed the hypothesis that LVSVi may serve as a novel prognostic marker in this often overlooked yet clinically important population.

## Data Availability

No datasets were generated or analysed during the current study.

## Abbreviations

AF: Atrial fibrillation
AUC: Area under the curve
CI: Confidence interval
ERO: Effective regurgitant orifice
HR: Hazard ratio
IQR: Interquartile range
LA: Left atrial
LAVi: Left atrial volume index
LV: Left ventricular
LVEF: Left ventricular ejection fraction
LVESD: Left ventricular end-systolic diameter
LVESDi: Left ventricular end-systolic diameter index
LVOT: Left ventricular outflow tract
LVSV: Left ventricular stroke volume
LVSVi: Left ventricular stroke volume index
MR: Mitral regurgitation
MV: Mitral valve
OR: Odds ratio
PISA: Proximal isovelocity surface area
ROC: Receiver operating characteristic
RVol: Regurgitant volume
VTI: Velocity-time integral
